# Effects of extended mixing processes on fresh, hardened and durable properties of cement systems incorporating fly ash

**DOI:** 10.1038/s41598-023-33312-x

**Published:** 2023-04-13

**Authors:** Issara Sereewatthanawut, Chinnapat Panwisawas, Chayut Ngamkhanong, Lapyote Prasittisopin

**Affiliations:** 1 Faculty of Engineering and Technology, Pathumthani University, Pathumthani, 12000 Thailand; 2 School of Engineering and Materials Science, Queen Mary University of London, London, E1 4NS UK; 3 Department of Civil Engineering, Faculty of Engineering, Chulalongkorn University, Bangkok, 10330 Thailand; 4 Department of Architecture, Faculty of Architecture, Chulalongkorn University, Bangkok, 10330 Thailand

**Keywords:** Environmental impact, Civil engineering

## Abstract

Specifications that correspond with system performance may guarantee the addition of value. Most specifications for ready-mixed concrete address limits on discharge time and truck-drum revolution counts. These limits have been developed for conventional concrete. As the uses of supplementary cementing materials (SCMs) become ubiquitous, it is important to determine whether these specifications are applicable to SCMs, that is, systems containing fly ash. This paper presents results of the effects of mixing time and mixer revolution counts on characteristics of lab-made pastes and mortars containing 20% and 50% fly ash. Their characteristics assessed include time-variant ion concentrations, setting time, flow, compressive strength, porosity, and apparent chloride diffusivity coefficient. Results indicate that with increasing mixing time and mixer revolution counts, mixtures with a replacement of fly ash exhibit improved both fresh and hardened characteristics. When mixed for 60 min or 25,505 revolution count, the 28-day compressive strengths of mixtures containing 20% and 50% fly ash are 50% to 100% higher than the neat cement. Fly ash is suggested to adopt in the extended mixing processes of cement systems.

## Introduction

Fly ash is a pozzolanic byproduct of the coal combustion process used to generate electricity. Currently, the coal combustion process accounts for roughly 50 to 55% of the United States' total energy generation^1,2^. Approximately 75 percent of the byproducts of this operation are fly ash^3–5^. Consequently, it is predicted that 500–550 million tons of fly ash are produced annually on a global scale^6,7^. Many sectors employ fly ash, including the agricultural and cement and concrete industries. It has been found that employing fly ash in the cement and concrete industries improves the performance characteristics of the hydrated products^8,9^. The main use of fly ash for the production of modern concrete composites, leading to new innovative solutions in this field, such as nano materials^10^, quaternary and ternary binders^11–13^, and active seeds^14,15^. The new innovative solutions can offer customized concrete products for various applications. However, more than 70 percent of the fly ash collected from power plants is not used, which poses a severe disposal challenge^16,17^. Coal-fired power facilities incur extra expenditures due to the disposal of fly ash. The annual cost is expected to be roughly $1.2 billion^18^. Therefore, more research and innovations that may expand the use of fly ash are necessary, particularly in the cement and concrete sectors. In addition to lowering disposal costs, this may enhance the performance characteristics of concrete mixes.

Concrete is the second most frequently utilized substance in the world, after water^19^. There have been significant efforts to limit CO_2_ emissions from the cement and concrete sectors due to environmental concerns. Nevertheless, the CO_2_ emissions from these businesses remain notably high, and more efforts are required. American Coal Ash Association (ACAA)^20^ calculated that using fly ash as a source of supplemental cementing material (SCM) in concrete can save CO_2_ emissions by 10 to 14 tons/year in the U.S. alone. Not only can the partial replacement of fly ash promote sustainability by lowering CO_2_ emissions, but it also decreases the expenses associated with producing concrete and disposing of fly ash. The Federal Highway Administration (FHWA) rules encourage concrete systems containing fly ash. This is especially true when the price of fly ash concrete is comparable to or lower than that of portland cement concrete (PCC)^21^. Hence, not all cement should be replaced with fly ash in a given mix. In addition to environmental and economic benefits, it is recognised that substituting Portland cement (PC) with fly ash improves the fresh properties and hardened performance of the hydrated product. As a pozzolanic material, calcium hydroxide (Ca(OH_2_)) may be reacted to produce calcium silicate hydrates that increase strength (C-S–H). These hydrates result in a densified interfacial transition zone (ITZ) and improved concrete microstructures at the interface of cement paste and aggregates^22,23^. Consequently, the performance of concrete systems incorporating fly ash may be superior to that of conventional concrete systems, and this may include ready-mixed concrete.

Ready-mixed concrete is defined by the American Society for Testing and Materials (ASTM) as concrete that is made and delivered to a client in a fresh state. Specifications for ready-mixed concrete from the American Association of State Highway and Transportation Officials (AASHTO), the American Concrete Institute (ACI), ASTM, and/or the State Highway Agencies (SHAs) address discharge time, truck-drum rotations, and/or concrete temperature limitations. In the United States, 48 of 50 SHAs restrict the duration before discharge to between 45 and 120 min; 30 of 50 SHAs limit the number of truck drum spins to between 250 and 320; and 45 of 50 SHAs limit the concrete temperature to between 28 and 38 °C. Because increasing discharge duration and number of drum revolutions might affect the workability of new cement and concrete mixes^24,25^, the majority of SHAs restrict these factors. Reduced workability makes it difficult to consolidate a new concrete. Improper placement and consolidation can lead to large voids, honeycombing, and increased permeability in hardened concrete^26–28^, resulting in a considerable decrease in compressive strength and durability. According to Anderson and Hodson^29^, the cost of polishing the hardened surface after casting is complete is about two to five times the cost of concrete's raw materials. Improved workability of fresh concrete can reduce placement efforts, resulting in lower construction expenses.

According to the Portland Cement Association (PCA)^30^, more than fifty percent of ready-mixed concrete contains fly ash. In infrastructure systems, fly ash-containing concretes are omnipresent. Although numerous research has evaluated the impacts of mixing factors on the performance of PCC, few studies have evaluated the effects of mixing variables on the performance characteristics of fly-ash-containing concrete.

Moreover, because PCC and concretes containing fly ash use the same limits of mixing time (i.e. 45 to 120 min) and total drum revolutions (i.e. 250 to 320 revolutions), contractors are questioning whether these current limits are still applicable to concretes containing fly ash and, if not, whether they should be modified. It is essential that the limit requirements adjust to new developments in materials and building processes, allowing for the addition of value. In addition, Hooton^31^ stated that limit requirements had to be changed to performance-based specifications so as not to restrict the development of alternative systems (e.g., systems containing fly ash). Before determining these difficulties, it is necessary to have a better knowledge of the performance characteristics of cementitious systems incorporating fly ash.

This research describes the laboratory assessment of the effects of mixing time and mixer revolution counts on the fresh and hardened properties of PC pastes and mortars containing 20% and 50% fly ash by weight. This research evaluates the time-dependent concentrations of hydroxyl, calcium, and aluminate ions in solution, the setting time of pastes, and the flowability of new mortars. The 1-, 7-, and 28-day compressive strengths (f_c_), 28-day porosity, and apparent chloride diffusion coefficient (*D*_*a*_) are tested as hardened properties of cement mortars.

## Materials and methods

### Materials

**Figure 1:**
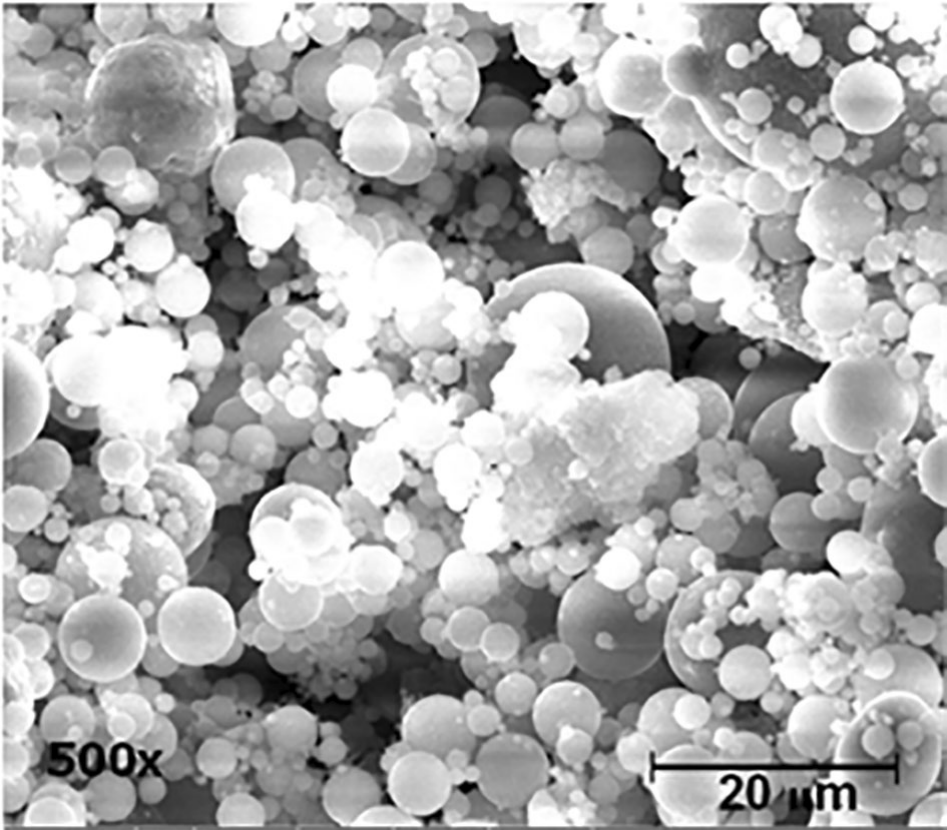
SEM micrograph of fly ash particles.

Type I PC was obtained from SCG, Thailand and used for all mixtures in this research. Class-F fly ash per ASTM C618 was procured from a local powerplant. The X-ray diffraction (XRD) chemical composition of PC and fly ash is shown in Table 1. A Scanning Electron Microscopy (SEM) image of fly ash particles is shown in Fig. 1. The particles of fly ash that were seen have a spherical form, a smooth surface, and a wide range of particle size distribution. Standard graded sand meeting ASTM C778 was used for the flowability, 28-day porosity, and *D*_*a*_ specimens. Type II de-ionized (DI) water (1 MΩ·cm at 25 °C) was used for all mixtures and experiments. Fine aggregate, used for the f_c_ specimens, was procured from a local source in Saraburi, Thailand and met the requirements of ASTM C33. The fineness modulus of the fine aggregate was 3.1 determined following ASTM C136*.* The specific gravity of the fine aggregate was 2.47 and the absorption was 3.08%. The specific gravity and absorption values were determined following ASTM C128.

**Table 1:** Chemical composition of PC and class-F fly ash.

Composition	Cement (%)	Fly ash (%)
SiO_2_	20.3	51.5
Al_2_O_3_	4.80	16.9
Fe_2_O_3_	3.50	6.20
MgO	0.70	4.10
SO_3_	2.80	0.70
CaO	63.9	11.7
Loss on ignition	2.60	0.25
Insoluble residue	0.11	–
Limestone	3.20	–
CaCO_3_ in limestone	97.8	–
Naeq	0.54	1.10

### Methods

#### Preparation method for cement pastes and mortars

The cement pastes and mortars were mixed in accordance with ASTM C305. The fly ash systems were prepared by substituting fly ash for cement by weight. A control combination (100% PC) was formulated and evaluated for comparison. The water-binder ratio (w/b) of the paste sample was 0.40. The specimens of mortar were made using a w/b ratio of 0.48 and a cement-fine aggregate ratio of 1.27:1. The C305 standard mandates two stages of mixing for pastes and mortars: low speed mixing (140 rpm) followed by intermediate speed mixing (250 and 285 rpm). Only the second stage's mixing time and speed were modified throughout this study. The mixing techniques for cement pastes and mortars are detailed in Table 2. Four mixing durations (2, 15, 60, and 90 min) and two mixing speeds (140 and 285 rpm) were investigated. This resulted in 210, 355, 2030, 4060, 8330, 12,600, 16,885, and 25,578 revolution counts for paste mixing and 350, 568, 2170, 4273, 8330, 12,600, 17,098, and 25,505 revolution counts for mortar mixing. All test results are based on triplicate tests.

**Table 2:** Mixing condition for cement pastes and mortars.

Mix. no	Paste				Mortar			
	First stage		Second stage		First stage		Second stage	
	Time (min)	Speed	Time (min)	Speed	Time (min)	Speed	Time (min)	Speed (rpm)
1	0.5	140	1.5	140	1	140	1	140
2	0.5	140	1.5	285	1	140	1	285
3	0.5	140	14.5	140	1	140	14	140
4	0.5	140	14.5	285	1	140	14	285
5	0.5	140	59.5	140	1	140	59	140
6	0.5	140	59.5	285	1	140	59	285
7*	0.5	140	89.5	140	1	140	89	140
8*	0.5	140	89.5	285	1	140	89	285

* for some tests.

#### Characterization methods

The time-variant hydroxyl ion concentrations in solution at early ages were evaluated using a pH electrode. The w/b value of the solutions was 4.0. Mixing for all systems was performed using a magnetic stirrer rotating at 0 and 400 rpm throughout the mixing period. Mixing at 0 rpm means the cement samples were manual mixed with water until they are uniform and then left without additional agitation. The time elapsed after introducing the unhydrated PC to the solution is referred to here as the “hydration time.” Solutions used for evaluating hydroxyl ion concentrations were analysed at 5, 10, 15, 30, 45, 60, 90, 120, 150, 180, 210, and 240 min (mixed at 0 and 400 rpm).

Using flame atomic absorption spectroscopy (FAAS), the amounts of aluminate and calcium ions were calculated. The process for determining hydroxyl ion concentrations was followed by mixing. At the same hydration durations as the studies of hydroxyl ion concentrations, solutions were evaluated at 300, 360, and 420 min of further hydration time.

At each hydration time, test solution (30 ml) was decanted from the mixing beaker and filtered using a vacuum pump and No. 40 filter paper. Ten ml of filtered solution was used for analysing aluminate ion concentrations and 1 ml of filtered solution was used for analysing calcium ion concentrations. Because high concentrations of calcium ions occur at early ages, filtered solutions for analysing calcium ion concentrations were diluted prior to the FAAS analyses. Filtered solutions for determining calcium ion concentrations were diluted with 9 ml of DI water to obtain the solution within the detection range of the FAAS. After decanting and diluting, 1 ml of lanthanum acid solution [50 g/l lanthanum oxide (La_2_O_3_) in 3 M hydrochloric acid (HCl)] was added to the solutions for the FAAS analyses. Concentrations of aluminate ions were determined using the FAAS with nitrous oxide-acetylene gas at a wavelength of 309.3 nm ignited at the temperature of 2600 to 2800˚C. The calcium concentrations were determined using air-acetylene gas at a wavelength of 422.7 nm ignited at the temperature of 2100 to 2400 ˚C. A blank sample (DI water only) was also analysed and used as a background correction.

Following ASTM C1437, the flowability of new mortars was evaluated. The setting time of cement pastes was evaluated using the ASTM C191 standard. Following ASTM C109, the 1-, 7-, and 28-day fc values were established. After casting, test specimens were stored for 24 h in plastic molds before being demolded. Before testing, deformed specimens were treated in a saturated lime solution. The 28-day porosity of mortars was measured using a modified ASTM C642 technique. Prasittisopin and Trejo^28^ detail methods for the modified porosity test. The D_a_ was calculated using ASTM C1556. After casting cylindrical mortar specimens measuring 75 mm by 150 mm to determine the D_a_, the specimens were stored in plastic molds for 24 h before being demolded. The specimens were subsequently subjected to a chloride solution for 35 days after being cured in saturated lime solution for 28 days. Specimens of powder were examined for chloride ion concentration in accordance with ASTM C1152. For testing chloride ion concentration, a computer-controlled potentiometric auto-titrator with a sample changer was utilised. After calculating the chloride ion concentration at various depths below the surface, the D_a_ was calculated using Fick's second rule, as indicated in Eq. 1.

1 $$ C(x,t) \, = \, C_{s} - \left[ {(C_{s} - C_{i} ) \, \times \, erf \, \left( {\frac{x}{{\sqrt {4D_{a} t} }}} \right)} \right] $$

where $C(x,t)$ is percent chloride ion concentration at depth *x* and exposed time *t*; *C*_*s*_ is predicted percent chloride ion concentration at the surface of the exposed mortar; *C*_*i*_ is percent initial chloride ion concentration of specimens before solution exposure; and *erf* is the error function.

#### Statistical data analysis

To compare sample means with two groups and more than two groups, respectively, the two-sample t-test and analysis of variances (ANOVA) were utilised. Before the analysis, the Shapiro–Wilk test was used to determine whether the data had a normal distribution, and the Levene’s test was used to investigate whether the data had an equal variance. The following statistical hypotheses were defined as:

2 $$\text{Null hypothesis }({H}_{0})\text{ : }{\mu }_{1}\hspace{0.33em}={\mu }_{2}\hspace{0.33em}=\hspace{0.33em}...\hspace{0.33em}=\hspace{0.33em}{\mu }_{a}$$

3 $$\text{Alternative hypothesis }({H}_{a})\hspace{0.33em}:\hspace{0.33em}{\mu }_{1}\hspace{0.33em}\ne \hspace{0.33em}{\mu }_{j}\hspace{0.33em}{\text{for}}\hspace{0.33em}{\text{some}}\hspace{0.33em}i\hspace{0.33em}\ne \hspace{0.33em}j$$

The 95% confidence intervals were used for all analyses. If the *H*_0_ is rejected (p-value ≤ 0.05), it is concluded that there is statistically significant difference at the 5% level between the means of group populations. Alternatively, if the *H*_0_ is not rejected (p-value > 0.05), it is concluded that there is no statistically significant difference at the 5% level between the means of group populations.

## Results and discussion

This section entails six experimental investigations including (1) ion concentration of hydroxyl, aluminate, and calcium, (2) flowability of fresh mixtures, (3) initial setting time, (4) compressive strengths at different curing times, (5) 28-d porosity, and (6) *D*_*a*_.

### Ion concentration

**Figure 2:**
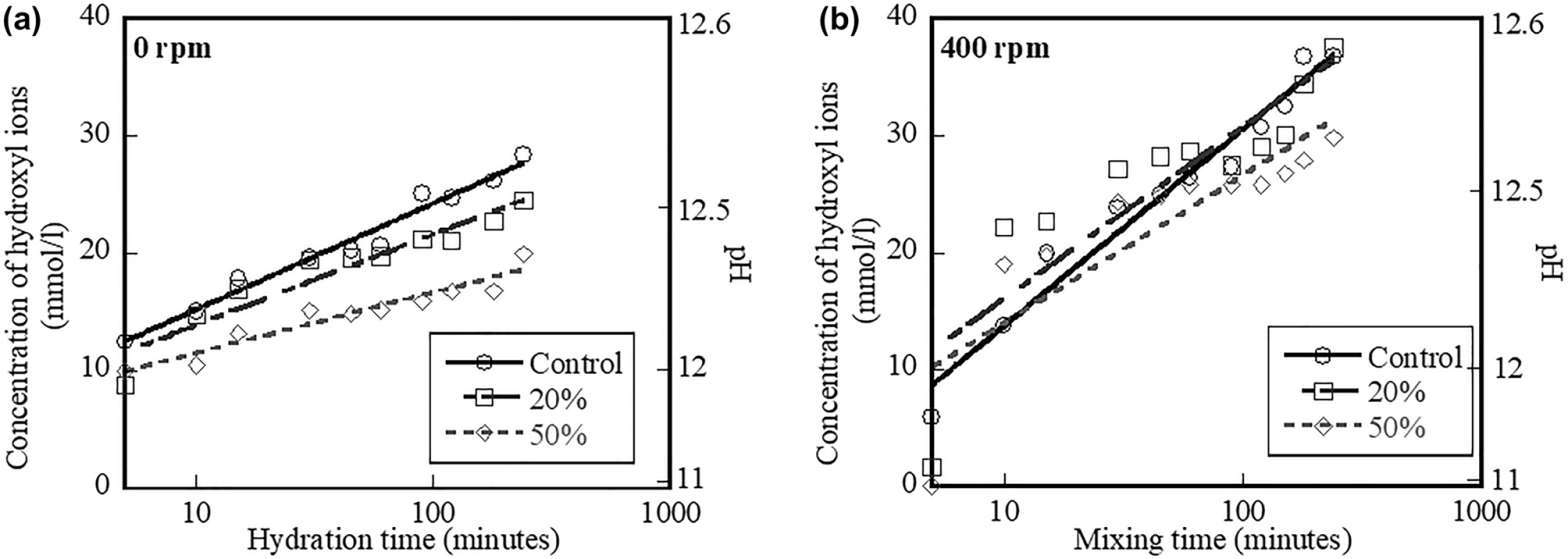
Effect of hydration time on hydroxyl ion concentration mixed at (**a**) 0 rpm and (**b**) 400 rpm of control system and systems containing 20% and 50% fly ash.

The effect of hydration time on the hydroxyl ion concentrations for the control, 20%, and 50% fly ash systems mixed at 0 and 400 rpm is shown Fig. 2a,b, respectively. Results indicate that the hydroxyl ion concentrations of all systems increase when hydration time increases. The slope of the fitted curve is referred to as the dissolution rate of the hydroxyl ions in solution. Larger slope indicates higher dissolution rate of hydroxyl ions in solution.

In Fig. 2a, the slopes of the control, 20%, and 50% fly ash systems are 9.0, 7.7, and 5.2 mmol/l/min, respectively. The larger slope of the control system corresponds with higher rate of dissolution of hydroxyl ions than the 20% and 50% fly ash systems. Increased percent replacement level of fly ash in the cementitious systems has been reported to result in decreased hydroxyl ion concentration in solution^32^. This is believed to occur because fly ash acts as “inert.” Hence, the fly ash mixtures contain less cement. Results in Fig. 2b are similar with the results in Fig. 2a, of which the slope of the control system mixed at 400 rpm (17 mmol/l/min) is larger than the slopes of the 20% (15 mmol/l/min), 50% fly ash (13 mmol/l/min) systems mixed at 400 rpm. Increased replacement level of fly ash results in decreased dissolution rates of hydroxyl ions, irrespective of mixing speed.

Comparing the results in Fig. 2b with the results in Fig. 2a indicates that the slopes of the control, 20%, and 50% fly ash systems mixed at 400 rpm are approximately 89%, 95%, and 150% higher than the slopes of the control, 20%, and 50% fly ash systems mixed at 0 rpm, respectively. The steeper slope is attributed to faster rate of dissolution of hydroxyl ions. This indicates that mixing speed strongly affects the dissolution rate of hydroxyl ions of all systems and could affect early-age characteristics of mixtures (as discussed later). Mixing speed increasingly affects the dissolution rate of hydroxyl ions in the systems containing higher replacement levels of fly ash.

**Figure 3:**
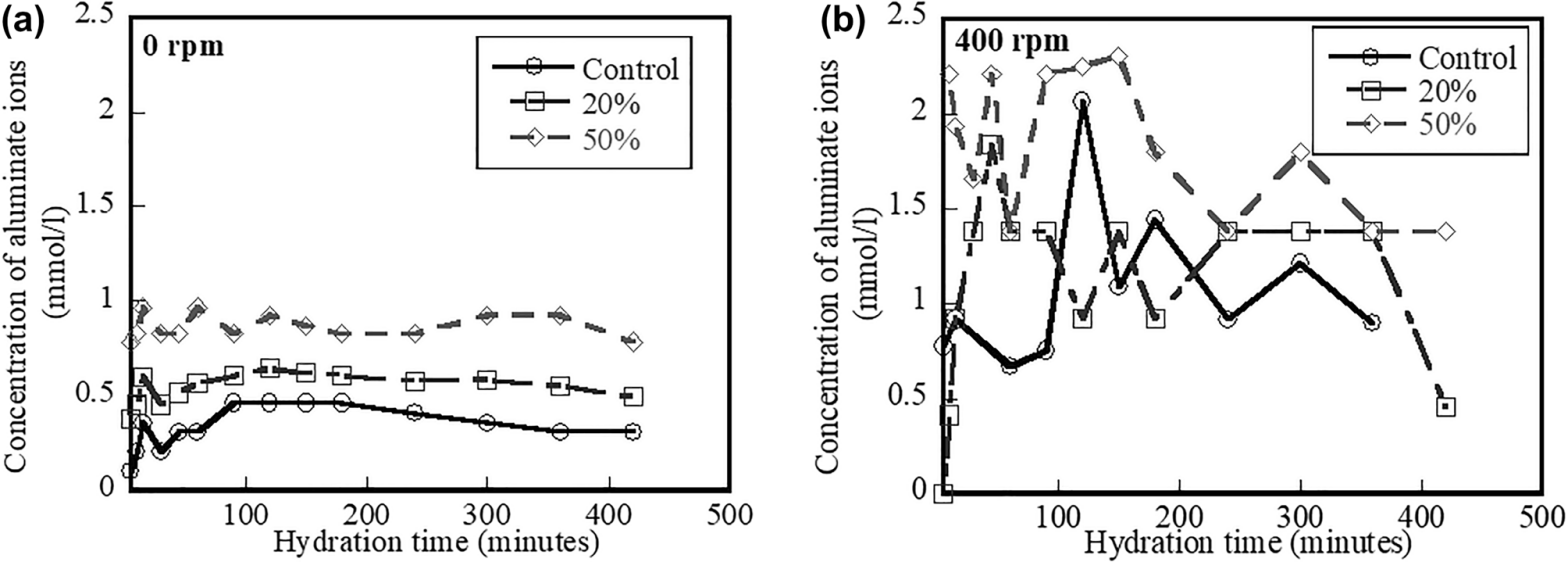
Effect of hydration time on aluminate ion concentration mixed at (**a**) 0 rpm and (**b**) 400 rpm of control system and systems containing 20% and 50% fly ash.

Figure 3a shows the time-variant concentrations of aluminate ions of the control, 20%, and 50% fly ash systems mixed at 0 rpm. Results indicate that the aluminate ion concentrations of the control system are lower than the aluminate ion concentrations of the 20% and 50% fly ash systems. The plot of aluminate ion concentrations of the control, 20%, and 50% fly ash systems mixed at 400 rpm as a function of the hydration time is shown in Fig. 3b. Like the results of all systems mixed at 0 rpm in Fig. 3a, the aluminate ion concentrations of the control system are lower than the 20% and 50% fly ash systems. More importantly, the aluminate ion concentrations mixed at 400 rpm for all systems are not stable, when compared with the aluminate ion concentrations in solutions mixed at 0 rpm. This “unstable” state likely occurs due to mixing speed. Because the aluminate ion concentrations are not stable in the systems mixed at 400 rpm, it is expected that there would be the differences of early-age characteristics (e.g., setting) between the systems that are continuously mixed and the systems that are mixed and then stop mixing.

**Figure 4:**
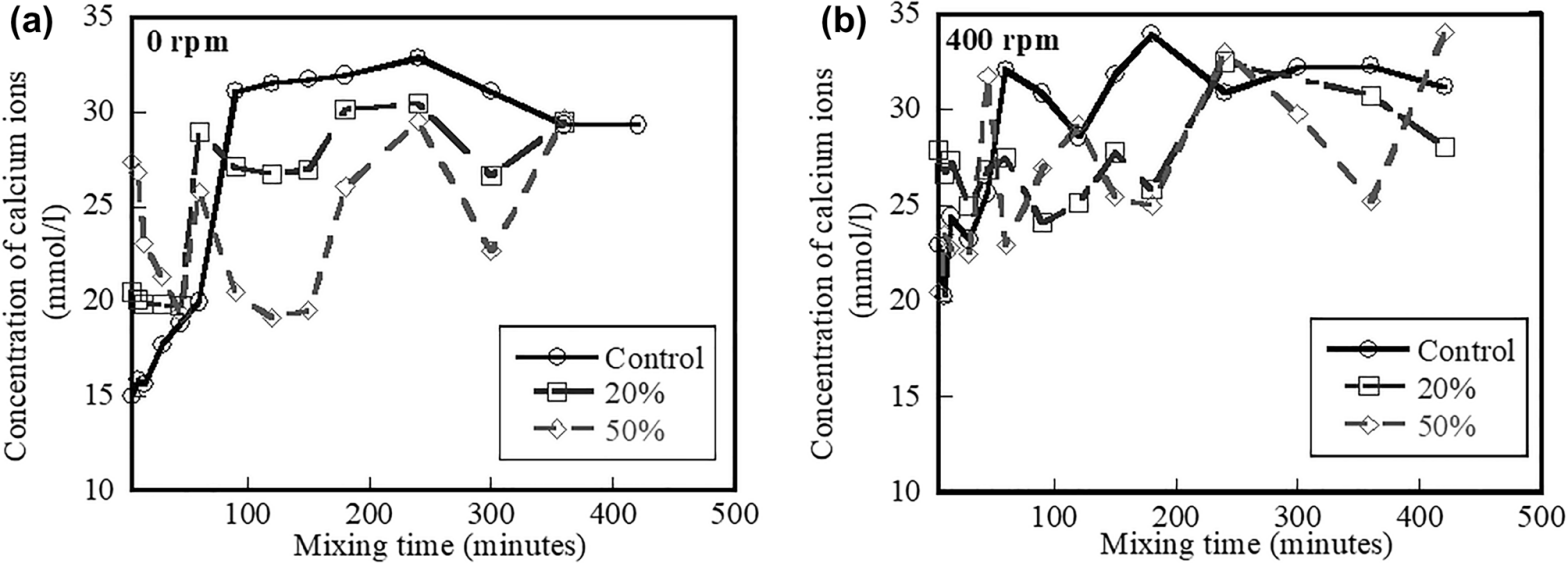
Effect of hydration time on calcium ion concentration mixed at (**a**) 0 rpm and (**b**) 400 rpm of control system and systems containing 20% and 50% fly ash.

The effect of hydration time on the calcium ion concentrations for the control, 20%, and 50% fly ash systems mixed at 0 and 400 rpm is shown Fig. 4a,b, respectively. Results in both figures indicate that the control systems mixed at 0 and 400 rpm have higher calcium ion concentrations than the 20% and 50% fly ash systems. The presence of fly ash results in reduced calcium ion concentrations at early ages due to less cement contents. In addition, these lower calcium ion concentrations could be a result of fly ash particles acting as nuclei for lime precipitation. Lawrence^33^ and Fraay et al*.*^34^ reported that the lime precipitation can occur when replacing cement with SCMs. Like the results of the aluminate ion concentrations in solutions mixed at different speeds (shown in Fig. 3a,b), the calcium ion concentrations in solutions mixed at 400 rpm are not stable compared to the solutions mixed at 0 rpm. Apparently, results from the aluminate and calcium ion concentrations indicate that when mixing continuously progresses, ions are likely unstable, thereby affecting other early-age characteristics.

Table 3 summarises the effects of the replacement of fly ash in cementitious systems on the concentrations of hydroxyl, aluminate, silicate, and calcium ions in solutions at early ages. Regarding the ion concentration studies for extended mixing processes, replacing cement with fly ash can lead to (1) decreased hydroxyl ion concentration due to less cement contents, (2) increased aluminate ion concentration due to a formation of aluminate-rich gel layers, (3) increased silicate ion concentration due to more silicon from fly ash in cementitious systems, and (4) decreased calcium ion concentration due to lime precipitation and less cement content^35,36^.

**Table 3:** Summary of ion concentration of cementitious containing fly ash.

Cementitious system	Ion concentration			
	Hydroxyl	Aluminate	Silicate	Calcium
With fly ash	↓	↑	↑	↓
Without fly ash	↑	↓	↓	↑
Possible cause	Less cement content	Gel formation	More silicon from fly ash	Lime precipitation/less cement content

### Flowability

**Figure 5:**
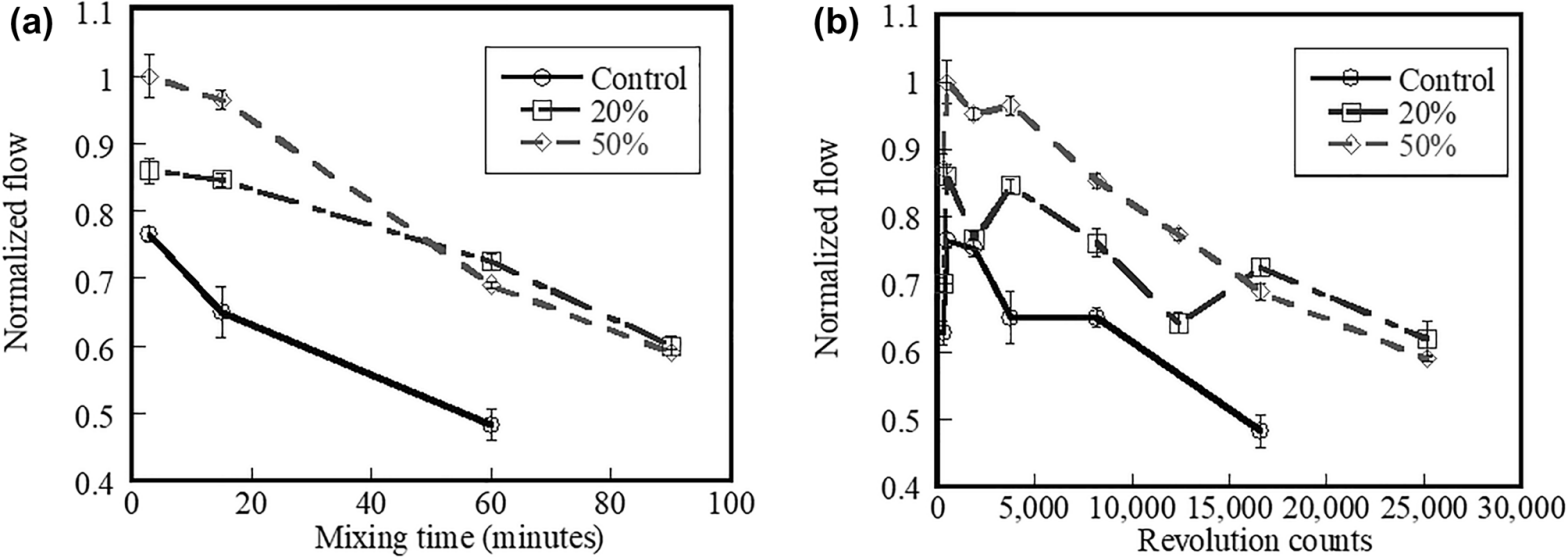
Effect of (**a**) mixing time and (**b**) mixer revolution counts on normalized flow of control system and systems containing 20% and 50% fly ash.

The effect of mixing time on normalised flowability of the control, 20%, and 50% fly ash systems mixed at 285 rpm is shown in Fig. 5a. The flow values are normalised with the maximum flow values (183 mm) of the 50% fly ash systems. Results indicate that a flow reduction of all systems is a result of increased mixing time. The flow of the fresh mortars of the 20% and 50% fly ash systems mixed for 2 min exhibits approximately 12% and 31% larger flow than the control system, respectively. The flow of the fresh mortars of the 20% and 50% fly ash systems mixed for 15 min exhibits approximately 30% and 48% larger flow than the control system, respectively. Lastly, the flow of the fresh mortars of the 20% and 50% fly ash systems mixed for 60 min exhibits approximately 50% and 43% larger flow than the control system, respectively. It is noted that the flowability in the figures has been tested at 285 rpm only. The lower mixing speed (140 rpm) was conducted for calculating different mixer revolution counts. Bentz and Ferraris^37^ reported that as hydrating products form, the early stiffening behavior is controlled by the gradual loss of free water from hydration reactions.

Paya et al*.*^38^ reported that flow of cementitious systems containing fly ash is influenced by several factors such as size distribution, morphology, surface condition, fineness, and loss on ignition of the fly ash particles. Replacing cement with fly ash is believed to make more water available for flow. Gopalan^39^ reported the water absorption characteristics of the cementitious systems are reduced when fly ash is present. In addition, the spherical particles and better particle size distribution of fly ash (which is shown in Fig. 1) lead to the reduction in friction between constituent particles in the systems (this commonly known as the “ball-ability” or “ball-bearing effect”)^40,41^. Thus, although increased mixing time results in reduced flow of the fly ash systems, the fresh mixtures can be mixed longer and still be castable due to improved flow.

The plot of the normalised flow of the control, 20%, and 50% fly ash systems as a function of mixer revolution counts is shown in Fig. 5b. Results indicate the flow of all systems significantly reduces with increasing mixer revolution counts. The control system has less flow than the 20% and 50% fly ash systems. Replacing cement with fly ash results in a significant increase in flow of the fresh mixture and that allows proper consolidation process of mixtures mixed at higher mixer revolution counts can be performed. Based on test data, specification’s limits on mixing time and mixer revolution counts may not be appropriated when fly ash is present in the cementitious systems.

### Setting time

**Figure 6:**
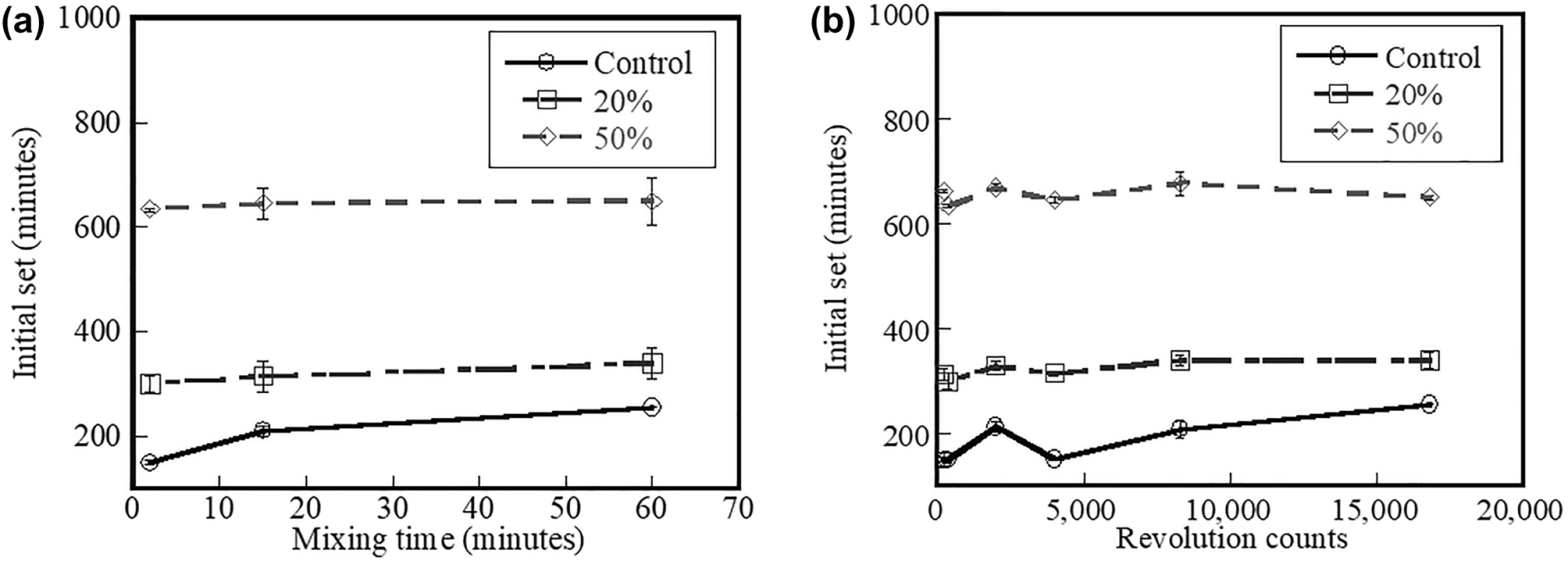
Effect of (**a**) mixing time and (**b**) mixer revolution counts on initial setting time of control system and systems containing 20% and 50% fly ash.

Figure 6a shows the effect of mixing time on the initial setting time of the control, 20%, and 50% fly ash systems. Results indicate that increased percent replacement level of fly ash results in delayed initial setting times. Possible reasons of delaying effect is likely due to the adsorption of calcium ions on the surface of fly ash^42^ and lowered hydroxyl ion concentrations. These lead to delayed nucleation and precipitation process of the Ca(OH)_2_, C-S–H, and ettringite. Results also indicate that increased mixing time leads to increased initial setting time of the control and 20% fly ash systems (ANOVA test with p-value < 0.05). This is likely because the ions in the systems are in the unstable state (as discussed) and the constituent’s particles are still deformed by a movement of mixing tools during continued mixing. This deformation of the particles likely disturbs the adhesion bonding of hydrating particles to form a larger structure. Hence, during mixing, the hydrating particles lose their load-supporting ability, consequently resulting in slower setting. After mixing discontinues, the adhesion bonding begins to form a larger structure and this structure, with time, begins to support external loads. However, it is assumed that part of water is consumed by hydration reactions during extended mixing since the hydration reactions can generate substantial amount of heat, and this heat of hydration leads to increased water evolution rate. Hence, less amount of water available for flow before casting and consolidating the specimens. This less amount of water of mixtures finally results in increased energy to cast and consolidate the specimens^28^. The hardened specimens seem to have higher void volume and larger void size. Voids in hardened cement systems generally results in lower fc and finally leads to deteriorate the durability and shorten serviceability. The porosity will be discussed later in Section "Compressive strength". It should be noted, however, that this work was done in paste and mortar systems while conducting research on either expanding the mix volume for the ready-mix concrete truck for each region or switching to concrete systems is mandatory.

Figure 6b show the effect of the initial setting time as a function of mixer revolution counts of the control, 20%, and 50% fly ash systems. According to the findings, it appears that the initial setting time tends to prolong as a function of the number of mixer revolutions. The initial setting times of cementitious systems containing fly ash are delayed as a result of increased mixing times as well as increased mixer revolution counts.

### Compressive strength

**Figure 7:**
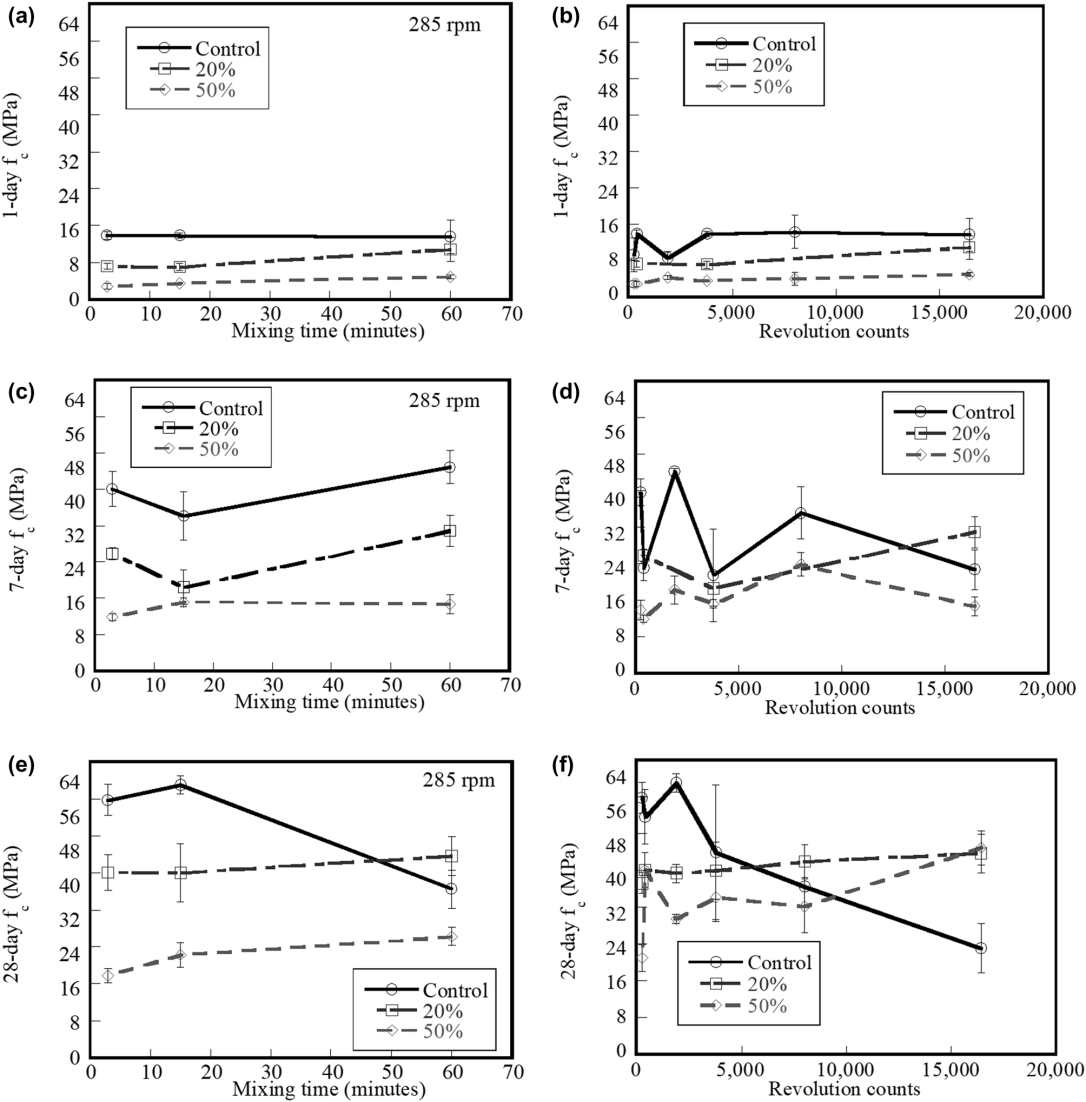
Effect of (**a**) mixing time and (**b**) mixer revolution counts on 1-day f_c_; (**c**) mixing time and (**d**) mixer revolution counts on 7-day f_c_; (**e**) mixing time and (**f**) mixer revolution counts on 28-day f_c_ of control system and systems containing 20% and 50% fly ash.

Figure 7a,b show the 1-day f_c_ of the control, 20%, and 50% fly ash systems as a function of mixing time and mixer revolution counts, respectively. Results indicate that increased percent replacement level of fly ash leads to lower 1-day compressive strength. Statistical analyses indicate that the 1-day f_c_ of all systems is not influenced by mixing time and mixer revolution counts (ANOVA test with p-value > 0.05).

Figure 7c,d show the effect of mixing time and mixer revolution counts on the 7-day f_c_ for the control system and systems containing 20% and 50% fly ash, respectively. Results indicate that the 7-day f_c_ of all systems is not significant affected by mixing time (ANOVA p-value > 0.05). In addition, the 7-day f_c_ of the control systems does not have a significant effect by mixing time (ANOVA p-value > 0.05); however, the 7-day f_c_ of the 20% and 50% fly ash does (ANOVA p-value < 0.05).

The effects of mixing time and mixer revolution counts on the 28-day f_c_ of the control, 20%, and 50% fly ash systems are shown in Fig. 7e,f, respectively. Similarly with the results of the 1- and 7-day f_c_, results indicate that mixing time does not a significant effect on 28-day f_c_ of all systems. In summary, extended mixing time does not influence on both early- and later-age f_c_. Regarding the compressive strength of ready-mixed concrete systems, their current discharge time limits as specified in many SHAs seem impractical use. However, here, the mixer revolution counts do influence 28-day f_c_. Increased mixer revolution counts result in a reduction of 28-day f_c_ of the control system but result in an increase of 28-day f_c_ of the systems containing fly ash. It has been reported that an increase in the consolidation energy consumption is responsible for the 28-day f_c_ drop that occurs as a function of the number of revolutions of the mixer^28^. The f_c_ will begin to decrease as soon as the energy available is insufficient to consolidate the specimens. The voids of cement systems enlarge and increase. As aforementioned, larger voids and greater void volumes of cement systems likely lead to reduced f_c_ and consequently lead to shorten their service life. When considering the revolution count, results apparently exhibit the SHAs’ limits should be in place. These can ensure the ready-mixed concrete can deliver to the users in good conditions. However, the systems that contain fly ash show an increase in 28-day f_c_, and it is assumed that this is because there is less water in their mixtures (which results in a lower w/b value). Increased rates of water evaporation and hydration reactions were claimed to be the cause of less water being present in the mixtures by Dewar and Anderson^43^.

Results herein indicate that mixing time does not have a significant influence on 1-, 7-, and 28-day f_c_. The summary of the influence of mixer revolution counts on the f_c_ of the control, 20%, and 50% fly ash systems is shown in Table 4. The early-age f_c_ values (1- and 7-day) do not have a significant impact by increasing the mixer revolution counts. On the other hand, the 28-day f_c_ will decrease for the control system as the number of mixer revolutions increases, but it will rise for the systems that include fly ash. Under the extended mixing conditions, the presence of fly ash in cementitious systems can improve the long-term compressive strength. Hence, the ready-mix concrete products should partially contain fly ash especially when a long haul is needed. As discussed before, the SHAs’ limits on discharge time and mixer revolution counts of ready-mixed concrete seems to require a revision when fly ash is present. In the revised versions, it is recommended that the longer discharge time and greater revolution count can be addressed, and this can be beneficial to the all stakeholders in ready-mixed concrete chain. The ready-mixed concrete producers can easily plan for various logistic routes and schedules. The consumers can receive a good quality of resulting concrete products. Finally, waste generated from over-limited ready-mixed concrete can be decreased that ultimately offer more cost effective and more sustainable manners to the industry^44^.

**Table 4:** Summary of the effect of mixer revolution count on the f_c_ of mixtures at different ages (↑ = increase; ↓ = decrease; and – = similar).

Age (days)	Effect of mixer revolution counts on the f_c_		
	Control	20% fly ash	50% fly ash
1	–	–	–
7	–	–	–
28	↓	↑	↑

### Porosity

**Figure 8:**
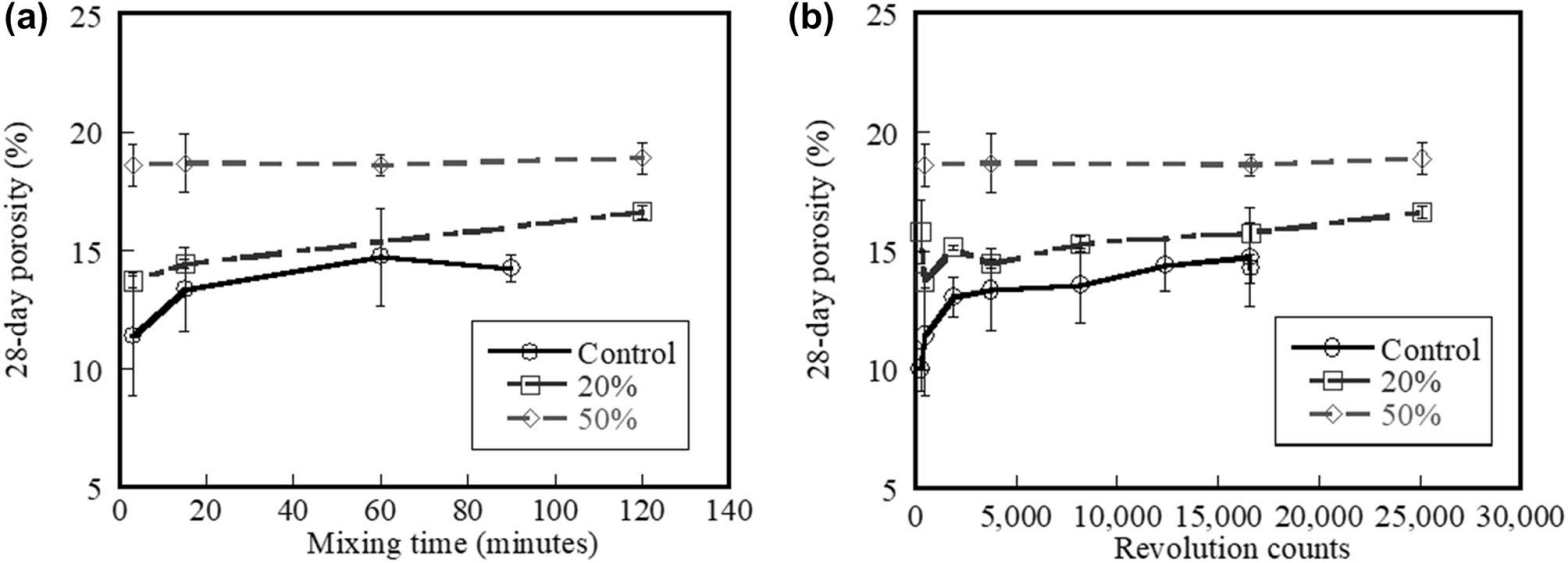
Effect of (**a**) mixing time and (**b**) mixer revolution counts on 28-day porosity of control system and systems containing 20% and 50% fly ash.

The effects of mixing time and mixer revolution counts on the 28-day porosity of the control, 20%, and 50% fly ash systems are shown in Fig. 8a,b, respectively. Results indicate that increased mixing time does not significantly affect the 28-day porosity for all systems (ANOVA p-value > 0.05). Results also reveal that significant increase in porosity should only relate to the control mix. Increased percent replacement level of fly ash results in higher porosity. Figure 8b shows that increasing the mixer revolution counts at lower revolutions (less than approximately 3000) results in a significant increase in porosity (ANOVA test with p-value = 0.013). However, increasing the mixer revolution counts at higher revolution counts (more than approximately 3000) has less influence on the porosity of mixtures (ANOVA test p-value > 0.05). The porosity of the systems containing fly ash does not exhibit this effect like the control system.

**Figure 9:**
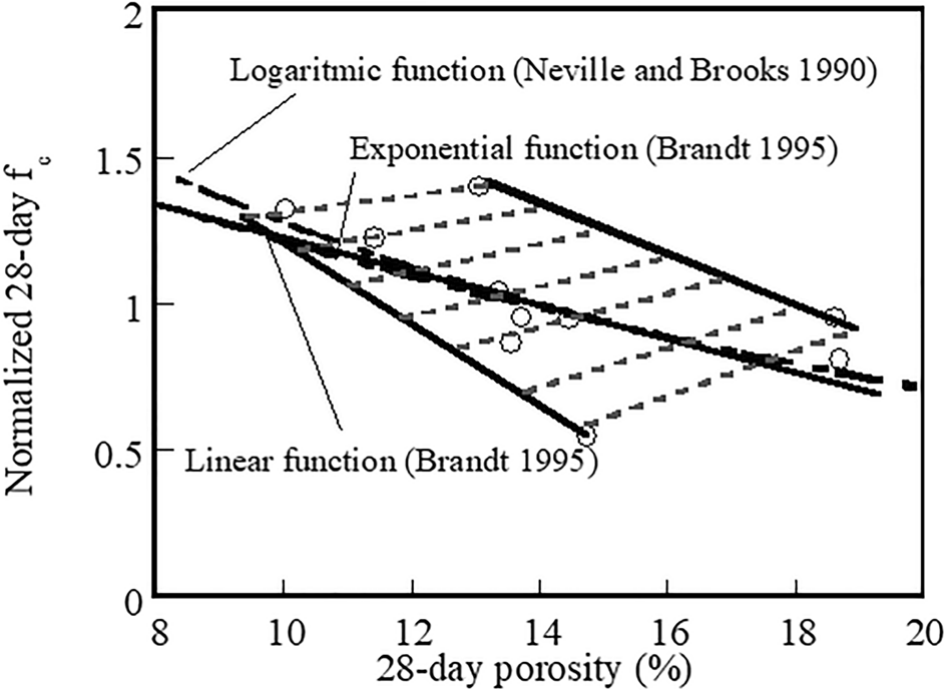
Relationship between normalized 28-day f_c_ and 28-day porosity of control systems and systems containing 20% and 50% fly ash mixed at different mixer revolution counts.

Figure 9 shows the correlation between the normalised 28-day compressive strength and the 28-day hardened porosity of the control, 20%, and 50% fly ash systems for different mixer revolution counts. The 28-day f_c_ values are normalised with the mean 28-day f_c_ for all mixtures. The linear fitted curve was illustrated in this study. The logarithmic fitted curve shown is reported by Neveille^45^ and the exponential and linear fitted curves are reported by Brandt^46^. Although different type of the fitted curve is not similar with the curves from previous studies, the linear fitted curve in this study is not distinguished from others. Results indicate that the 28-day f_c_ is reduced with increasing porosity and this increased porosity is likely due to reduced flow caused by higher mixer revolution counts. It is assumed that the primary criteria reflecting the overall performance of macrostructures are parameters relating to the adequate flow of fresh cement mixtures resulting from extended mixing.

### Apparent chloride diffusivity

**Figure 10:**
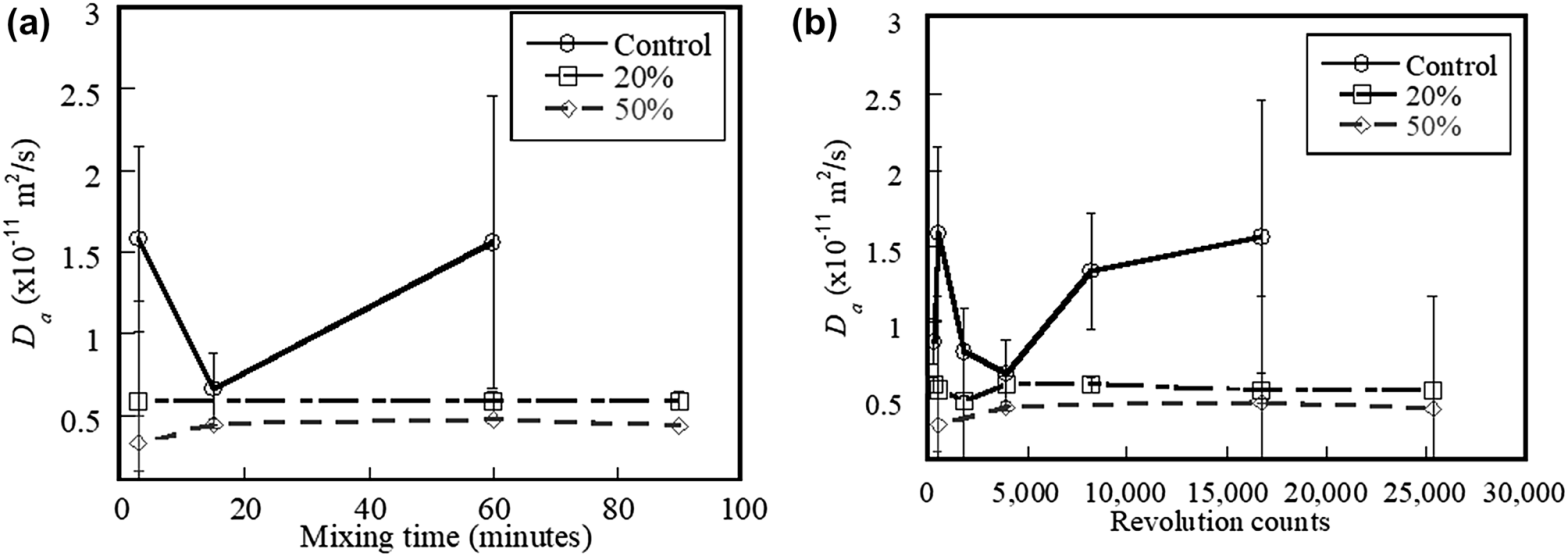
Effect of (**a**) mixing time and (**b**) mixer revolution counts on *D*_*a*_ of control system and systems containing 20% and 50% fly ash.

Figure 10a,b show the effects of mixing time and mixer revolution counts on the *D*_*a*_ of the control, 20%, and 50% fly ash systems, respectively. Results indicate that neither mixing time nor mixer revolution counts has a significant effect on the *D*_*a*_ for all systems (ANOVA p-value > 0.05). The control system exhibits higher *D*_*a*_ than the systems containing fly ash (ANOVA p-value = 0.026). Consequently, only the influence of material components influences corrosion resistance, not mixing activities. It is common knowledge that adding fly ash to cement systems can result in a denser microstructure and reduced porosity at later ages. This is because pozzolanic reactions (reaction between CaO and S to form C-S–H products) progress at later ages^47,48^. Golewski^47^ mentioned that the homogenous and uniform structure of the portland cement system containing FA was seen after 14-day curing period, which resulted from the transformation of disordered phases into compact and homogenous forms and the filling of porous voids of C-S–H phase. Sabet et al*.*^49^ reported that the presence of fly ash in the cementitious systems can react with Ca(OH)_2_ to produce C-S–H products and also bind chloride ions by the aluminate phases during chloride exposed period. These lead to reduced transport rates, and finally the service life of concrete structure can be extended.

## Conclusions

This study assessed the influence of extended mixing processes on the performance parameters of cementitious systems including fly ash. At various mixing periods and mixing speeds, the dissolution kinetics of hydroxyl, calcium, and aluminate ions were examined. At various mixing periods and mixing revolution counts, the fresh and hardened characteristics of pastes and mortars were examined. The findings suggested that:

- Increased mixing speed resulted in increased dissolution kinetics of hydroxyl ion in solutions and made calcium and aluminate ions stay in the unstable state.
- Increased replacement level of fly ash leads to increased flow values but slower setting time. Thus, while longer mixing processes lower the flow values, fly ash mixes can be castable due to better flow.
- With increasing mixer revolution counts, the 28-day f_c_ of systems with no fly ash reduce but the 28-day f_c_ of the systems with fly ash increase.
- The porosity of the fly ash mortars does not influenced by mixing time and mixer revolution counts.
- The D_a_ is not affected by these mixing time and mixer revolution counts. However, when fly ash presents, the D_a_ can be affected by these mixing time and mixer revolution counts.

Existing requirements from the majority of SHAs may not be relevant to cementitious systems including fly ash, based on the study's findings about mixing time and mixer revolution counts. Ongoing research examines the influence of these present constraints on the performance qualities of concrete containing fly ash.

## Data Availability

The datasets used and/or analysed during the current study available from the corresponding author on reasonable request.
